# *m*-Xylylenediaminium dinitrate

**DOI:** 10.1107/S1600536814004620

**Published:** 2014-03-05

**Authors:** Sofian Gatfaoui, Hassouna Dhaouadi, Thierry Roisnel, Mohamed Rzaigui, Houda Marouani

**Affiliations:** a Laboratoire de Chimie des Matériaux, Faculté des Sciences de Bizerte, 7021 Zarzouna Bizerte, Tunisia; b Laboratoire des Matériaux Utiles, Institut National de Recherche et d’Analyse Physico-chimique, Pole Technologique de Sidi-Thabet, 2020 Tunis, Tunisia; c Centre de Diffractométrie X, UMR 6226 CNRS, Unité Sciences Chimiques de Rennes, Université de Rennes I, 263 Avenue du Général Leclerc, 35042 Rennes, France

## Abstract

The asymmetric unit of the title salt, C_8_H_14_N_2_^2+^·2NO_3_^−^, contains two independent dications and four independent nitrate anions. The crystal structure consists of discrete nitrate ions, three of which stack in layers parallel to (001) at *z* = 0 and 0.5. These layers are connected *via m*-xylylenediaminium dications. The fourth anion is sandwiched by the two independent organic cations in the asymmetric unit. In the crystal, the ions are connected by a large number of bifurcated and non-bifurcated N—H⋯O(O) hydrogen bonds, forming sheets parallel to (100). These sheets are connected by C—H⋯O hydrogen bonds, forming a three-dimensional network.

## Related literature   

For related nitrate salts, see: Gatfaoui *et al.* (2013 ▶, 2014 ▶); Marouani *et al.* (2012 ▶); Kefi *et al.* (2013 ▶). For the dichloride salt of the title cation, see: Cheng & Li (2008 ▶). For background to hydrogen bonding and aromatic π–π stacking inter­actions, see: Brown (1976 ▶); Blessing (1986 ▶); Janiak (2000 ▶).
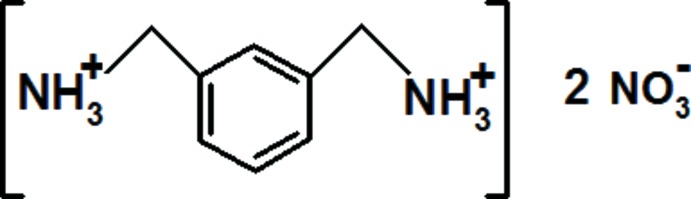


## Experimental   

### 

#### Crystal data  

C_8_H_14_N_2_^2+^·2NO_3_^−^
*M* *_r_* = 262.23
Monoclinic, 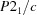

*a* = 21.4308 (7) Å
*b* = 5.7255 (2) Å
*c* = 20.4476 (5) Å
β = 108.502 (1)°
*V* = 2379.28 (13) Å^3^
*Z* = 8
Mo *K*α radiation
μ = 0.13 mm^−1^
*T* = 150 K
0.47 × 0.24 × 0.17 mm

#### Data collection  

Bruker APEXII diffractometer
Absorption correction: multi-scan (*SADABS*; Bruker, 2006 ▶) *T*_min_ = 0.889, *T*_max_ = 0.979
19108 measured reflections
5457 independent reflections
4429 reflections with *I* > 2σ(*I*)
*R*_int_ = 0.036

#### Refinement  

*R*[*F* ^2^ > 2σ(*F* ^2^)] = 0.047
*wR*(*F* ^2^) = 0.129
*S* = 1.04
5457 reflections
329 parameters
H-atom parameters constrained
Δρ_max_ = 0.51 e Å^−3^
Δρ_min_ = −0.28 e Å^−3^

### 

Data collection: *APEX2* (Bruker, 2006 ▶); cell refinement: *SAINT* (Bruker, 2006 ▶); data reduction: *SAINT*; program(s) used to solve structure: *SIR97* (Altomare *et al.*, 1999 ▶); program(s) used to refine structure: *SHELXL97* (Sheldrick, 2008 ▶); molecular graphics: *ORTEP-3 for Windows* (Farrugia, 2012 ▶) and *DIAMOND* (Brandenburg & Putz, 2005 ▶); software used to prepare material for publication: *WinGX* (Farrugia, 2012 ▶) and *CRYSCAL* (T. Roisnel, local program).

## Supplementary Material

Crystal structure: contains datablock(s) I. DOI: 10.1107/S1600536814004620/bh2495sup1.cif

Structure factors: contains datablock(s) I. DOI: 10.1107/S1600536814004620/bh2495Isup2.hkl

Supporting information file. DOI: 10.1107/S1600536814004620/bh2495Isup3.cml

CCDC reference: 989215

Additional supporting information:  crystallographic information; 3D view; checkCIF report

## Figures and Tables

**Table 1:** Hydrogen-bond geometry (Å, °)

*D*—H⋯*A*	*D*—H	H⋯*A*	*D*⋯*A*	*D*—H⋯*A*
N1—H1*A*⋯O3	0.91	2.06	2.9545 (16)	168
N1—H1*B*⋯O6	0.91	2.34	2.9872 (18)	128
N1—H1*B*⋯O9^i^	0.91	2.23	2.9554 (17)	137
N1—H1*C*⋯O2^i^	0.91	2.29	3.197 (2)	173
N1—H1*C*⋯O3^i^	0.91	2.42	3.0837 (16)	130
N2—H2*B*⋯O10^ii^	0.91	2.48	3.032 (2)	119
N2—H2*B*⋯O12^ii^	0.91	1.96	2.8391 (19)	162
N2—H2*C*⋯O10^iii^	0.91	1.92	2.829 (2)	173
N2—H2*C*⋯O11^iii^	0.91	2.51	3.074 (2)	120
N3—H3*A*⋯O5	0.91	2.42	3.0008 (17)	122
N3—H3*A*⋯O6	0.91	1.91	2.8155 (16)	175
N3—H3*B*⋯O4^iv^	0.91	1.93	2.7974 (17)	159
N3—H3*B*⋯O6^iv^	0.91	2.50	2.9910 (16)	114
N3—H3*C*⋯O3	0.91	2.03	2.8897 (19)	157
N4—H4*A*⋯O1	0.91	2.35	3.079 (2)	137
N4—H4*A*⋯O2	0.91	2.15	2.9967 (19)	155
N4—H4*B*⋯O8^v^	0.91	2.42	3.0673 (16)	128
N4—H4*B*⋯O9^v^	0.91	2.12	2.9368 (17)	150
N4—H4*C*⋯O7^iii^	0.91	2.52	3.2192 (18)	134
N4—H4*C*⋯O8^iii^	0.91	1.99	2.8810 (16)	165
C7—H7⋯O2^i^	0.95	2.53	3.309 (2)	140
C8—H8*A*⋯O12^vi^	0.99	2.46	3.373 (2)	153
C9—H9*B*⋯O4^vii^	0.99	2.31	3.267 (2)	163
C16—H16*A*⋯O4^iii^	0.99	2.33	3.267 (2)	157

Symmetry codes: (i) 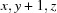; (ii) 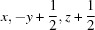; (iii) 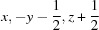; (iv) 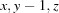; (v) 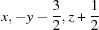; (vi) 
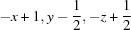
; (vii) 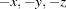.

**Fig. 1.**
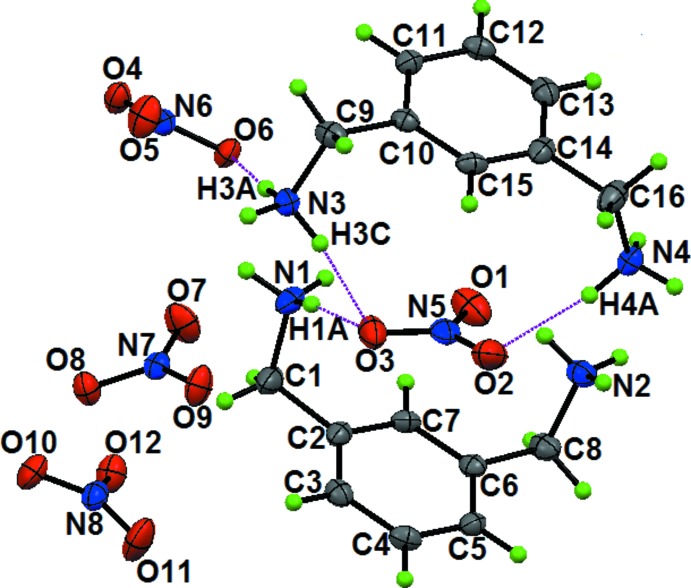
An ORTEP view of (I) with displacement ellipsoids drawn at the 30% probability level. H atoms are represented as small spheres of arbitrary radii. Hydrogen bonds are shown as dotted lines.

**Fig. 2.**
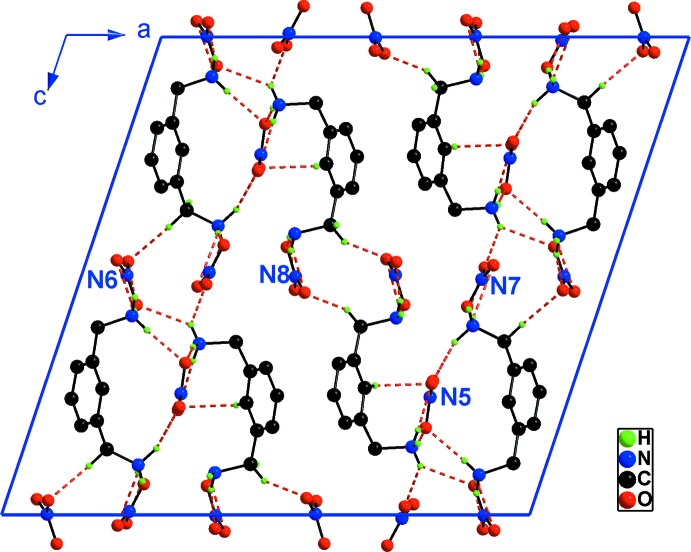
Projection of (I) along the b axis. The H-atoms not involved in H-bonding are omitted.

## supplementary crystallographic information

### 1.1. Synthesis and crystallization

An aqueous solution containing 4 mmol of HNO_3_ in 10 ml of water was added to 2 mmol of *m*-xylylenedi­amine in 10 ml of water. The obtained solution was stirred for 1 h and then left to stand at room temperature. Colorless single crystals of the title compound were obtained after one week.

### 1.2. Refinement

Crystal data, data collection and structure refinement details are summarized in Table 1. All H atoms bonded to C and N atoms were treated as riding with C—H = 0.99 Å (methyl­ene) or 0.95 Å (aromatic CH), N—H = 0.91 Å (NH_3_), and with *U*_iso_(H) = 1.2*U*_eq_(C) and *U*_iso_(H) = 1.5*U*_eq_(N).

### 2. Results and discussion

As a part of our study of crystal packing containing the nitrate anion (Marouani *et al.*, 2012; Gatfaoui *et al.*, 2013, 2014; Kefi *et al.*, 2013), we report here the preparation and the structural investigation of a new organic nitrate, (C_8_H_14_N_2_)·(NO_3_)_2_ (I). The asymmetric unit of (I) is composed of two *m*-xylylenediaminium dications and four nitrate anions (Fig. 1). The structure of the compound consists of discrete nitrate ions stacked in layers parallel to the (001) plane at *z* = 0 and 1/2, for N6/O4/O5/O6, N7/O7/O8/O9, N8/O10/O11/O12 and at *z* = 0.25 and 0.75 for N5/O1/O2/O3, separated by organic cations. Each organic entity is connected to different nitrate layers at *z* = 0 and 1/2. At the same time, the N5O_3_ nitrate groups ensure the cohesion between the organic groups (Fig. 2). The structural cohesion is established by a three-dimensional network of N—H···O and weak C—H···O hydrogen bonds.

Inter­atomic bond lengths and angles of the nitrate anions spread respectively within the ranges 1.2314 (19)–1.2719 (17) Å and 118.69 (15)–121.48 (13)°. These geometrical features have also been noticed in other related crystal structures (Gatfaoui *et al.*, 2013, 2014).

In this crystal arrangement two independent *m*-xylylediaminium cations are present. Both ammonium groups in each cation adopt a *cis* conformation with respect to the benzene ring. The *trans* conformation has been observed in C_8_H_14_N_2_^2+^·2Cl^-^ (Cheng & Li, 2008). Thus, the cation conformation is modified when substituting nitrate anions by chlorides. Examination of the organic cations shows that the bond distances and angles show no significant differences from those obtained in other compounds involving the same organic groups (Cheng & Li, 2008). The aromatic rings are planar with an average deviation of 0.0022 Å and form a dihedral angle of 3.85° in the asymmetric unit. The inter-planar distance between nearby benzene rings in the crystal structure is in the vicinity of 4.59 Å, which is much longer than 3.80 Å, value required for the formation of π–π inter­actions (Janiak, 2000).

The established weak H-bonds (Brown, 1976; Blessing, 1986) of types N—H···O and C—H···O involve oxygen atoms of the nitrate anions as acceptors, and the protonated nitro­gen atoms and carbon atoms of *m*-xylylenediaminium as donors.

### Crystal data

**Table:**

C_8_H_14_N_2_^2+^·2NO_3_^−^	*F*(000) = 1104
*M**_r_* = 262.23	*D*_x_ = 1.464 Mg m^−^^3^
Monoclinic, *P*2_1_/*c*	Mo *K*α radiation, λ = 0.71073 Å
Hall symbol: -P 2ybc	Cell parameters from 6173 reflections
*a* = 21.4308 (7) Å	θ = 3.0–27.3°
*b* = 5.7255 (2) Å	µ = 0.13 mm^−^^1^
*c* = 20.4476 (5) Å	*T* = 150 K
β = 108.502 (1)°	Prism, colourless
*V* = 2379.28 (13) Å^3^	0.47 × 0.24 × 0.17 mm
*Z* = 8	

### Data collection

**Table:**

Bruker APEXII diffractometer	4429 reflections with *I* > 2σ(*I*)
Graphite monochromator	*R*_int_ = 0.036
CCD rotation images, thin slices scans	θ_max_ = 27.5°, θ_min_ = 3.0°
Absorption correction: multi-scan (*SADABS*; Bruker, 2006)	*h* = −26→22
*T*_min_ = 0.889, *T*_max_ = 0.979	*k* = −6→7
19108 measured reflections	*l* = −27→27
5457 independent reflections	

### Refinement

**Table:**

Refinement on *F*^2^	Primary atom site location: structure-invariant direct methods
Least-squares matrix: full	Secondary atom site location: difference Fourier map
*R*[*F*^2^ > 2σ(*F*^2^)] = 0.047	Hydrogen site location: inferred from neighbouring sites
*wR*(*F*^2^) = 0.129	H-atom parameters constrained
*S* = 1.04	*w* = 1/[σ^2^(*F*_o_^2^) + (0.0626*P*)^2^ + 1.0374*P*] where *P* = (*F*_o_^2^ + 2*F*_c_^2^)/3
5457 reflections	(Δ/σ)_max_ = 0.003
329 parameters	Δρ_max_ = 0.51 e Å^−^^3^
0 restraints	Δρ_min_ = −0.28 e Å^−^^3^
0 constraints	

### Fractional atomic coordinates and isotropic or equivalent isotropic displacement parameters (Å^2^)

**Table:**

	*x*	*y*	*z*	*U*_iso_*/*U*_eq_	
N1	0.26601 (6)	0.0292 (2)	0.14220 (7)	0.0272 (3)	
H1A	0.2554	−0.1199	0.1498	0.041*	
H1B	0.2374	0.0818	0.1018	0.041*	
H1C	0.2635	0.1223	0.1774	0.041*	
C1	0.33450 (8)	0.0351 (3)	0.13828 (9)	0.0329 (4)	
H1D	0.3490	0.1991	0.1376	0.040*	
H1E	0.3356	−0.0416	0.0953	0.040*	
C2	0.38017 (7)	−0.0900 (3)	0.19976 (8)	0.0221 (3)	
C3	0.40757 (7)	−0.3050 (3)	0.19264 (8)	0.0236 (3)	
H3	0.3998	−0.3709	0.1481	0.028*	
C4	0.44632 (7)	−0.4234 (3)	0.25056 (9)	0.0229 (3)	
H4	0.4656	−0.5688	0.2453	0.027*	
C5	0.45719 (7)	−0.3312 (3)	0.31598 (8)	0.0215 (3)	
H5	0.4827	−0.4153	0.3555	0.026*	
C6	0.43055 (7)	−0.1149 (3)	0.32352 (8)	0.0198 (3)	
C7	0.39272 (7)	0.0044 (3)	0.26535 (8)	0.0208 (3)	
H7	0.3751	0.1530	0.2704	0.025*	
C8	0.43934 (8)	−0.0113 (3)	0.39380 (9)	0.0278 (4)	
H8A	0.4758	−0.0919	0.4288	0.033*	
H8B	0.4508	0.1561	0.3938	0.033*	
N2	0.37725 (7)	−0.0370 (3)	0.41183 (7)	0.0270 (3)	
H2A	0.3426	0.0137	0.3760	0.041*	
H2B	0.3802	0.0495	0.4500	0.041*	
H2C	0.3713	−0.1899	0.4204	0.041*	
N3	0.12089 (6)	−0.4473 (2)	0.08345 (7)	0.0215 (3)	
H3A	0.1231	−0.2923	0.0746	0.032*	
H3B	0.1162	−0.5304	0.0442	0.032*	
H3C	0.1585	−0.4920	0.1166	0.032*	
C9	0.06349 (8)	−0.4918 (3)	0.10789 (9)	0.0267 (4)	
H9A	0.0552	−0.6620	0.1076	0.032*	
H9B	0.0239	−0.4169	0.0759	0.032*	
C10	0.07523 (7)	−0.3980 (3)	0.17964 (8)	0.0195 (3)	
C11	0.04955 (7)	−0.1832 (3)	0.19040 (8)	0.0205 (3)	
H11	0.0256	−0.0906	0.1521	0.025*	
C12	0.05893 (7)	−0.1046 (3)	0.25702 (9)	0.0234 (3)	
H12	0.0410	0.0413	0.2642	0.028*	
C13	0.09441 (8)	−0.2383 (3)	0.31318 (8)	0.0248 (3)	
H13	0.1005	−0.1838	0.3587	0.030*	
C14	0.12113 (7)	−0.4520 (3)	0.30315 (8)	0.0242 (3)	
C15	0.11106 (7)	−0.5290 (3)	0.23635 (8)	0.0216 (3)	
H15	0.1291	−0.6747	0.2291	0.026*	
C16	0.15782 (8)	−0.6046 (4)	0.36295 (10)	0.0368 (4)	
H16A	0.1348	−0.6021	0.3979	0.044*	
H16B	0.1575	−0.7675	0.3466	0.044*	
N4	0.22723 (6)	−0.5286 (2)	0.39575 (7)	0.0227 (3)	
H4A	0.2492	−0.5372	0.3644	0.034*	
H4B	0.2470	−0.6234	0.4322	0.034*	
H4C	0.2279	−0.3787	0.4108	0.034*	
N5	0.26305 (6)	−0.4703 (3)	0.24641 (7)	0.0287 (3)	
O1	0.26758 (7)	−0.2898 (3)	0.28036 (7)	0.0436 (4)	
O2	0.26855 (6)	−0.6677 (3)	0.27431 (7)	0.0415 (3)	
O3	0.25175 (6)	−0.4589 (2)	0.18215 (6)	0.0350 (3)	
N6	0.08581 (6)	0.0558 (2)	−0.00143 (7)	0.0236 (3)	
O4	0.07924 (6)	0.2525 (2)	−0.03009 (6)	0.0283 (3)	
O5	0.05713 (7)	−0.1176 (2)	−0.03262 (6)	0.0391 (3)	
O6	0.12308 (5)	0.03715 (19)	0.06066 (6)	0.0246 (3)	
N7	0.23961 (6)	−0.4784 (2)	−0.00724 (7)	0.0219 (3)	
O7	0.22667 (7)	−0.3051 (2)	0.02227 (7)	0.0402 (3)	
O8	0.24994 (6)	−0.4545 (2)	−0.06438 (6)	0.0290 (3)	
O9	0.24162 (6)	−0.6782 (2)	0.01751 (6)	0.0346 (3)	
N8	0.40614 (7)	0.0407 (3)	0.00192 (7)	0.0279 (3)	
O10	0.37062 (6)	0.0189 (2)	−0.06046 (6)	0.0332 (3)	
O11	0.43303 (8)	−0.1319 (3)	0.03493 (7)	0.0481 (4)	
O12	0.41292 (6)	0.2396 (2)	0.02899 (6)	0.0347 (3)	

### Atomic displacement parameters (Å^2^)

**Table:**

	*U*^11^	*U*^22^	*U*^33^	*U*^12^	*U*^13^	*U*^23^
N1	0.0203 (6)	0.0296 (8)	0.0274 (7)	0.0020 (5)	0.0012 (5)	0.0040 (6)
C1	0.0228 (8)	0.0458 (11)	0.0301 (9)	0.0040 (7)	0.0082 (7)	0.0153 (8)
C2	0.0159 (7)	0.0263 (8)	0.0237 (8)	−0.0021 (6)	0.0056 (6)	0.0068 (6)
C3	0.0215 (7)	0.0271 (8)	0.0233 (8)	−0.0056 (6)	0.0086 (6)	−0.0042 (7)
C4	0.0222 (7)	0.0159 (7)	0.0332 (9)	−0.0007 (6)	0.0126 (6)	−0.0017 (6)
C5	0.0173 (7)	0.0213 (8)	0.0257 (8)	0.0015 (6)	0.0063 (6)	0.0059 (6)
C6	0.0152 (6)	0.0218 (7)	0.0226 (7)	−0.0026 (6)	0.0062 (5)	−0.0019 (6)
C7	0.0167 (7)	0.0163 (7)	0.0307 (8)	0.0012 (5)	0.0093 (6)	0.0023 (6)
C8	0.0214 (7)	0.0342 (9)	0.0277 (8)	−0.0031 (7)	0.0076 (6)	−0.0080 (7)
N2	0.0245 (7)	0.0318 (8)	0.0266 (7)	0.0001 (6)	0.0106 (6)	−0.0073 (6)
N3	0.0225 (6)	0.0219 (7)	0.0205 (6)	−0.0001 (5)	0.0073 (5)	−0.0005 (5)
C9	0.0220 (7)	0.0303 (9)	0.0290 (9)	−0.0045 (6)	0.0100 (6)	−0.0094 (7)
C10	0.0167 (7)	0.0191 (7)	0.0240 (8)	−0.0035 (5)	0.0083 (6)	−0.0023 (6)
C11	0.0177 (7)	0.0185 (7)	0.0247 (8)	−0.0015 (5)	0.0061 (6)	0.0030 (6)
C12	0.0213 (7)	0.0187 (7)	0.0328 (9)	−0.0016 (6)	0.0121 (6)	−0.0045 (7)
C13	0.0224 (7)	0.0298 (8)	0.0232 (8)	−0.0072 (6)	0.0089 (6)	−0.0041 (7)
C14	0.0167 (7)	0.0282 (8)	0.0266 (8)	−0.0044 (6)	0.0053 (6)	0.0089 (7)
C15	0.0169 (7)	0.0161 (7)	0.0329 (9)	0.0000 (5)	0.0095 (6)	0.0016 (6)
C16	0.0233 (8)	0.0447 (11)	0.0379 (10)	−0.0055 (8)	0.0032 (7)	0.0207 (9)
N4	0.0243 (6)	0.0205 (6)	0.0205 (7)	0.0010 (5)	0.0033 (5)	0.0015 (5)
N5	0.0175 (6)	0.0420 (9)	0.0270 (7)	−0.0025 (6)	0.0077 (5)	−0.0016 (7)
O1	0.0410 (8)	0.0502 (9)	0.0449 (8)	−0.0155 (6)	0.0210 (6)	−0.0169 (7)
O2	0.0359 (7)	0.0463 (8)	0.0471 (8)	0.0113 (6)	0.0200 (6)	0.0128 (7)
O3	0.0266 (6)	0.0554 (8)	0.0228 (6)	−0.0075 (6)	0.0077 (5)	−0.0010 (6)
N6	0.0241 (7)	0.0254 (7)	0.0209 (7)	0.0023 (5)	0.0067 (5)	−0.0019 (6)
O4	0.0364 (6)	0.0253 (6)	0.0220 (6)	0.0069 (5)	0.0073 (5)	0.0029 (5)
O5	0.0484 (8)	0.0304 (7)	0.0296 (7)	−0.0083 (6)	−0.0002 (6)	−0.0073 (6)
O6	0.0269 (6)	0.0243 (6)	0.0186 (5)	0.0021 (4)	0.0013 (4)	−0.0001 (5)
N7	0.0217 (6)	0.0213 (7)	0.0212 (7)	−0.0024 (5)	0.0046 (5)	−0.0005 (5)
O7	0.0586 (9)	0.0290 (7)	0.0412 (7)	−0.0008 (6)	0.0275 (7)	−0.0099 (6)
O8	0.0389 (7)	0.0267 (6)	0.0250 (6)	−0.0006 (5)	0.0152 (5)	0.0014 (5)
O9	0.0478 (8)	0.0246 (6)	0.0266 (6)	−0.0043 (5)	0.0051 (5)	0.0072 (5)
N8	0.0237 (7)	0.0338 (8)	0.0251 (7)	−0.0016 (6)	0.0063 (6)	0.0087 (6)
O10	0.0302 (6)	0.0347 (7)	0.0261 (6)	−0.0003 (5)	−0.0034 (5)	0.0037 (5)
O11	0.0600 (9)	0.0416 (8)	0.0322 (7)	0.0123 (7)	−0.0005 (6)	0.0127 (6)
O12	0.0406 (7)	0.0324 (7)	0.0282 (6)	−0.0058 (5)	0.0070 (5)	0.0025 (5)

### Geometric parameters (Å, º)

**Table:**

N1—C1	1.495 (2)	C9—H9B	0.9900
N1—H1A	0.9100	C10—C15	1.390 (2)
N1—H1B	0.9100	C10—C11	1.393 (2)
N1—H1C	0.9100	C11—C12	1.388 (2)
C1—C2	1.507 (2)	C11—H11	0.9500
C1—H1D	0.9900	C12—C13	1.389 (2)
C1—H1E	0.9900	C12—H12	0.9500
C2—C7	1.391 (2)	C13—C14	1.393 (2)
C2—C3	1.392 (2)	C13—H13	0.9500
C3—C4	1.388 (2)	C14—C15	1.386 (2)
C3—H3	0.9500	C14—C16	1.506 (2)
C4—C5	1.387 (2)	C15—H15	0.9500
C4—H4	0.9500	C16—N4	1.489 (2)
C5—C6	1.393 (2)	C16—H16A	0.9900
C5—H5	0.9500	C16—H16B	0.9900
C6—C7	1.388 (2)	N4—H4A	0.9100
C6—C8	1.510 (2)	N4—H4B	0.9100
C7—H7	0.9500	N4—H4C	0.9100
C8—N2	1.496 (2)	N5—O1	1.2314 (19)
C8—H8A	0.9900	N5—O2	1.255 (2)
C8—H8B	0.9900	N5—O3	1.2599 (18)
N2—H2A	0.9100	N6—O4	1.2566 (17)
N2—H2B	0.9100	N6—O5	1.2334 (18)
N2—H2C	0.9100	N6—O6	1.2719 (17)
N3—C9	1.489 (2)	N7—O7	1.2379 (18)
N3—H3A	0.9100	N7—O8	1.2637 (17)
N3—H3B	0.9100	N7—O9	1.2459 (17)
N3—H3C	0.9100	N8—O10	1.2671 (18)
C9—C10	1.507 (2)	N8—O11	1.2321 (19)
C9—H9A	0.9900	N8—O12	1.2542 (19)
			
C1—N1—H1A	109.5	N3—C9—C10	111.42 (12)
C1—N1—H1B	109.5	N3—C9—H9A	109.3
H1A—N1—H1B	109.5	C10—C9—H9A	109.3
C1—N1—H1C	109.5	N3—C9—H9B	109.3
H1A—N1—H1C	109.5	C10—C9—H9B	109.3
H1B—N1—H1C	109.5	H9A—C9—H9B	108.0
N1—C1—C2	109.48 (13)	C15—C10—C11	119.09 (14)
N1—C1—H1D	109.8	C15—C10—C9	119.82 (14)
C2—C1—H1D	109.8	C11—C10—C9	121.08 (14)
N1—C1—H1E	109.8	C12—C11—C10	120.02 (14)
C2—C1—H1E	109.8	C12—C11—H11	120.0
H1D—C1—H1E	108.2	C10—C11—H11	120.0
C7—C2—C3	119.06 (14)	C11—C12—C13	120.23 (15)
C7—C2—C1	119.72 (15)	C11—C12—H12	119.9
C3—C2—C1	121.14 (15)	C13—C12—H12	119.9
C4—C3—C2	120.05 (14)	C12—C13—C14	120.34 (15)
C4—C3—H3	120.0	C12—C13—H13	119.8
C2—C3—H3	120.0	C14—C13—H13	119.8
C5—C4—C3	120.59 (15)	C15—C14—C13	118.81 (14)
C5—C4—H4	119.7	C15—C14—C16	119.58 (16)
C3—C4—H4	119.7	C13—C14—C16	121.55 (16)
C4—C5—C6	119.72 (14)	C14—C15—C10	121.51 (14)
C4—C5—H5	120.1	C14—C15—H15	119.2
C6—C5—H5	120.1	C10—C15—H15	119.2
C7—C6—C5	119.42 (14)	N4—C16—C14	112.69 (13)
C7—C6—C8	119.01 (14)	N4—C16—H16A	109.1
C5—C6—C8	121.52 (14)	C14—C16—H16A	109.1
C6—C7—C2	121.13 (14)	N4—C16—H16B	109.1
C6—C7—H7	119.4	C14—C16—H16B	109.1
C2—C7—H7	119.4	H16A—C16—H16B	107.8
N2—C8—C6	110.10 (12)	C16—N4—H4A	109.5
N2—C8—H8A	109.6	C16—N4—H4B	109.5
C6—C8—H8A	109.6	H4A—N4—H4B	109.5
N2—C8—H8B	109.6	C16—N4—H4C	109.5
C6—C8—H8B	109.6	H4A—N4—H4C	109.5
H8A—C8—H8B	108.2	H4B—N4—H4C	109.5
C8—N2—H2A	109.5	O1—N5—O2	121.35 (15)
C8—N2—H2B	109.5	O1—N5—O3	119.95 (15)
H2A—N2—H2B	109.5	O2—N5—O3	118.69 (15)
C8—N2—H2C	109.5	O4—N6—O6	118.86 (13)
H2A—N2—H2C	109.5	O5—N6—O4	121.10 (13)
H2B—N2—H2C	109.5	O5—N6—O6	120.04 (13)
C9—N3—H3A	109.5	O7—N7—O9	121.48 (13)
C9—N3—H3B	109.5	O7—N7—O8	119.70 (13)
H3A—N3—H3B	109.5	O9—N7—O8	118.80 (13)
C9—N3—H3C	109.5	O11—N8—O12	121.20 (14)
H3A—N3—H3C	109.5	O11—N8—O10	119.97 (15)
H3B—N3—H3C	109.5	O12—N8—O10	118.83 (14)
			
N1—C1—C2—C7	−67.34 (19)	N3—C9—C10—C15	−82.48 (18)
N1—C1—C2—C3	109.35 (17)	N3—C9—C10—C11	98.88 (16)
C7—C2—C3—C4	0.6 (2)	C15—C10—C11—C12	−1.1 (2)
C1—C2—C3—C4	−176.12 (14)	C9—C10—C11—C12	177.56 (14)
C2—C3—C4—C5	1.2 (2)	C10—C11—C12—C13	0.6 (2)
C3—C4—C5—C6	−1.8 (2)	C11—C12—C13—C14	0.3 (2)
C4—C5—C6—C7	0.8 (2)	C12—C13—C14—C15	−0.7 (2)
C4—C5—C6—C8	178.08 (14)	C12—C13—C14—C16	−177.88 (14)
C5—C6—C7—C2	1.0 (2)	C13—C14—C15—C10	0.1 (2)
C8—C6—C7—C2	−176.39 (13)	C16—C14—C15—C10	177.42 (14)
C3—C2—C7—C6	−1.7 (2)	C11—C10—C15—C14	0.7 (2)
C1—C2—C7—C6	175.10 (14)	C9—C10—C15—C14	−177.94 (13)
C7—C6—C8—N2	73.72 (19)	C15—C14—C16—N4	102.05 (19)
C5—C6—C8—N2	−103.59 (16)	C13—C14—C16—N4	−80.8 (2)

### Hydrogen-bond geometry (Å, º)

**Table:**

*D*—H···*A*	*D*—H	H···*A*	*D*···*A*	*D*—H···*A*
N1—H1*A*···O3	0.91	2.06	2.9545 (16)	168
N1—H1*B*···O6	0.91	2.34	2.9872 (18)	128
N1—H1*B*···O9^i^	0.91	2.23	2.9554 (17)	137
N1—H1*C*···O2^i^	0.91	2.29	3.197 (2)	173
N1—H1*C*···O3^i^	0.91	2.42	3.0837 (16)	130
N2—H2*B*···O10^ii^	0.91	2.48	3.032 (2)	119
N2—H2*B*···O12^ii^	0.91	1.96	2.8391 (19)	162
N2—H2*C*···O10^iii^	0.91	1.92	2.829 (2)	173
N2—H2*C*···O11^iii^	0.91	2.51	3.074 (2)	120
N3—H3*A*···O5	0.91	2.42	3.0008 (17)	122
N3—H3*A*···O6	0.91	1.91	2.8155 (16)	175
N3—H3*B*···O4^iv^	0.91	1.93	2.7974 (17)	159
N3—H3*B*···O6^iv^	0.91	2.50	2.9910 (16)	114
N3—H3*C*···O3	0.91	2.03	2.8897 (19)	157
N4—H4*A*···O1	0.91	2.35	3.079 (2)	137
N4—H4*A*···O2	0.91	2.15	2.9967 (19)	155
N4—H4*B*···O8^v^	0.91	2.42	3.0673 (16)	128
N4—H4*B*···O9^v^	0.91	2.12	2.9368 (17)	150
N4—H4*C*···O7^iii^	0.91	2.52	3.2192 (18)	134
N4—H4*C*···O8^iii^	0.91	1.99	2.8810 (16)	165
C7—H7···O2^i^	0.95	2.53	3.309 (2)	140
C8—H8*A*···O12^vi^	0.99	2.46	3.373 (2)	153
C9—H9*B*···O4^vii^	0.99	2.31	3.267 (2)	163
C16—H16*A*···O4^iii^	0.99	2.33	3.267 (2)	157

Symmetry codes: (i) *x*, *y*+1, *z*; (ii) *x*, −*y*+1/2, *z*+1/2; (iii) *x*, −*y*−1/2, *z*+1/2; (iv) *x*, *y*−1, *z*; (v) *x*, −*y*−3/2, *z*+1/2; (vi) −*x*+1, *y*−1/2, −*z*+1/2; (vii) −*x*, −*y*, −*z*.
